# Constructing Memory, Imagination, and Empathy: A Cognitive Neuroscience Perspective

**DOI:** 10.3389/fpsyg.2012.00576

**Published:** 2013-01-09

**Authors:** Brendan Gaesser

**Affiliations:** ^1^Department of Psychology, Harvard UniversityCambridge, MA, USA; ^2^Center for Brain Science, Harvard UniversityCambridge, MA, USA

**Keywords:** episodic memory, episodic simulation, mental simulation, imagination, functional magnetic resonance imaging, empathy, prosocial behavior

## Abstract

Studies on memory, imagination, and empathy have largely progressed in isolation. Consequently, humans’ empathic tendencies to care about and help other people are considered independent of our ability to remember and imagine events. Despite this theoretical autonomy, work from across psychology, and neuroscience suggests that these cognitive abilities may be linked. In the present paper, I tentatively propose that humans’ ability to vividly imagine specific events (as supported by constructive memory) may facilitate prosocial intentions and behavior. Evidence of a relationship between memory, imagination, and empathy comes from research that shows imagination influences the perceived and actual likelihood an event occurs, improves intergroup relations, and shares a neural basis with memory and empathy. Although many questions remain, this paper outlines a new direction for research that investigates the role of imagination in promoting empathy and prosocial behavior.

## Introduction

Every day there are families with an inept cat marooned in a tree, friends with a couch to be moved, neighbors’ homes damaged by a storm, pedestrians hit by a car: people in need of help. To the sufferers’ benefit, other people tend to provide assistance, sometimes at a great cost to personal welfare (Hein et al., 2010). Partly accounting for why such selfless behaviors exist, some researchers have proposed humans’ rampant evolutionary success is due to our species’ ability to understand, collaborate with, and help others (Tomasello, 2000; Nowak and Highfield, 2011). Contributing to these abilities is our capacity for empathy. A term used to describe various concepts in the social psychology literature, empathy is a multifaceted construct that has included vicariously experiencing another person’s emotions (affect-sharing), deliberately considering another person’s perspective in order to understand their thoughts and feelings (mentalizing), and a desire to improve another person’s welfare (prosocial concern; Zaki and Ochsner, 2012). For the purposes of this paper, I use empathy to imply the latter construct, viewing the former two as means of – but not always – eliciting empathy and prosocial behavior (Batson, 1991).

There are several factors known to modulate willingness to help (e.g., closeness of a relationship, social norms), but predominate among them is mentalizing the perspective of the person in need. Adopting the other person’s perspective focuses our attention on how he or she is affected by an adverse situation, eliciting an empathic feeling that then increases the perspective-taker’s willingness to help (Batson, 1991; see, Epley et al., 2006; Barber et al., 2010; for mentalizing conditions that elicit egotism). But that might not always be the case. In contrast to mentalizing, a path to facilitate helping that is less reliant on emotional concern may come from episodic simulation, the ability to vividly imagine specific personal events. While mentalizing and episodic simulation are related in that both processes consist of a mental shift away from the immediate environment and into hypothetical experiences (Buckner and Carroll, 2007), the content of those hypothetical experiences differs across these processes. Whereas mentalizing involves inferring mental states from another person’s perspective, episodic simulation involves imagining scenarios specific in time and place from a first-person perspective.

In recent years, the functional role of memory has extended beyond remembering the past to include imagining the future (Addis et al., 2007; Schacter et al., 2008; Szpunar, 2010). One prominent theory, the *constructive episodic simulation hypothesis*, posits that the cognitive raw materials of imagined future experiences are bits and bobs of episodic memories (Schacter and Addis, 2007, 2009). The flexibility of constructive memory promotes the recombination of details gleaned across past episodic experiences into novel representations of future events that allow individuals to readily confront previously un-encountered situations (Schacter et al., 2008; Buckner, 2010). The adaptive function of imagining events is thought to mainly derive from the opportunity to “test out” one or more versions of what might happen by simulating the outcome of anticipated future events. Simulation reduces the cost of engaging in behavior while supporting planning and prediction, which enables humans to learn from simulated missteps without actually lifting a foot (Buckner and Carroll, 2007; Gilbert and Wilson, 2007; Schacter and Addis, 2007; see, Schacter, 2012 for a comprehensive review). Here, I offer an additional function for episodic simulation that has not previously been considered: facilitating socially desirable actions.

In what follows, I selectively piece together work from across the cognitive and social domains of psychology and neuroscience, marshaling support for the role of episodic simulation in understanding and helping others. First, I will review studies that demonstrate imagination’s effect on the perceived likelihood that an event will happen, an effect that is likely to extend to imagined helping. Second, I will then look at recent promising work that demonstrates imagination’s positive impact on empathy biases against dissimilar others. Third, I will briefly examine the neural basis of episodic simulation and empathy by highlighting overlapping brain regions and contemplate the shared cognitive processes these regions may support. Finally, I highlight what I think will be productive avenues for future research exploring episodic simulation’s contribution to empathy.

## Imagining Makes it So

Relief that people in need will receive is partially dependent on the extent that others are willing to offer assistance; the more willing someone is to help, the more likely the person in need will gain relief. This point seems obvious, but it is easy to mistake self-evidence for inconsequence. While prosocial behavior is pervasive it is by no means assured (Batson et al., 1997). Evaluating one’s willingness to help in any meaningful way requires a person to reflect on the perceived probability that he or she will help someone. Although there is currently no direct evidence that imagining helping increases willingness to help, there is relevant, though mostly disconnected, work from the memory and social judgment literatures.

Memory researchers have long been interested in the fallible nature of memory, attempting to understand why memories are imperfect re-constructions, rather than literal reproductions, of past experiences (Bartlett, 1932; Schacter, 2001). In the mid-1990s, studies on imagination inflation showed that imagining a novel event increased the perceived likelihood that it occurred in one’s past, and in some cases led to rich false memories of experiences that never occurred (Hyman and Pentland, 1996; Garry and Polaschek, 2000). Less interested in past events, social psychologists have found that imagining hypothetical events increases the perceived probability that the event will occur in the future (Carroll, 1978; Anderson, 1983; Greenwald et al., 1987). Examining decision-making heuristics, Carroll (1978) initially demonstrated imagination’s influence on predictions. Prior to the 1976 presidential election, subjects imagined events related to either a Ford or Carter victory and later were instructed to predict the likelihood that either candidate would win. The candidate that subjects imagined winning was rated more likely to win.

More recent work has shown this effect for simulating specific future social experiences (e.g., a family gathering, job interview, or first date). Simulating the same experience multiple times increased estimates of perceived plausibility for future experiences. Further, ratings of simulated detail, ease, and emotional intensity tracked with plausibility (Szpunar and Schacter, 2012). Although relatively little is known about the neural basis of these imagination inflation effects, tentative evidence from related work on imagination suggests that activity in the precuneus may drive increased plausibility ratings (Weiler et al., 2010). Thus, imagining (possibly mediated by the precuneus) makes it so, or at least makes it *seem* so.

Critical to assessing the potential contribution of imagination to helping others is to determine imagination’s influence on actual behavior. A recent study on voting intentions found that imagination influenced self-perceptions (including perceived probability of voting), which subsequently influenced the likelihood that the subject would vote a day later (Libby et al., 2007). Consistent with the facilitating effect of imagined behavior on actual behavior, other researchers found that imagination increased estimated willingness to subscribe to a local cable company immediately following imagining the benefits and services the company offered. Strikingly, 2–3 months later, imagination-inflated estimates predicted actual subscriptions (Gregory et al., 1982, experiment 4).

Currently, it remains unknown whether imagining helping others will increase our willingness to help and subsequent helping behavior. However, based on research from cognitive and social psychology showing imagination’s facilitating effect on perceived probability across a variety of situations, the effect will likely extend to prosocial behavior. Despite an absence of research on imagination inflation and empathy, a good deal is known about the positive effect of imagination on intergroup relations, a topic I turn to next.

## Imagination Fosters Positive Intergroup Relations

Human helping behavior is pervasive, but this empathic response to alleviate another person’s suffering is diminished or absent when the sufferer is a member of a different social, racial, or cultural group (Cuddy et al., 2007; Cikara et al., 2011). To the betterment of intergroup relations, however, several techniques have been shown to reduce prejudice and empathy directed at outgroup members (Batson and Ahmad, 2009). One particular effective technique for improving intergroup attitudes and reducing prejudice involves simply imagining a positive interaction with an outgroup member (Crisp and Turner, 2009).

Imagined social contact offers a flexible and relatively minimal manipulation that can elicit robust prosocial change. For example, young subjects who spent 1 min imagining a positive interaction with an elderly person showed reduced ingroup bias compared to participants who imagined an outdoor scene or merely thought about an outgroup member. Imagined contact reduced anxiety about interacting with an outgroup member and increased willingness to work with outgroup members (Turner et al., 2007). Imagined contact may even promote intentions to engage in future contact (Husnu and Crisp, 2010), and reduce implicit prejudice (Turner and Crisp, 2010).

While imagined contact is a useful technique for improving intergroup relations, it is unlikely to be as effective as face-to-face contact (Turner et al., 2007). Part of the reason for this difference may be that direct perception leads to relevant knowledge being more cognitively available than imagined events (Tversky and Kahneman, 1973). Perhaps the closer an imagined event approximates a genuine percept (i.e., the more coherent, vivid, and detailed an event in the mind’s eye) the greater its cognitive and behavioral sway would be (Anderson, 1983; Crisp et al., 2010). Therefore, if one could boost the vividness and detail of an imagined intergroup interaction, then perhaps the prosocial effectiveness of imagined contact would be enhanced.

Husnu and Crisp (2010) hypothesized that more elaborate imagined contact would enhance intentions of future contact with outgroup members and that subjective ratings of vividness would mediate this enhancement. Elaboration was manipulated by instructing subjects to specifically envision when and where they would come into contact with an imagined outgroup member (i.e., high elaboration condition) in contrast with the previously used imagined contact condition that did not require subjects to specify when and where (i.e., low elaboration condition). The researchers found that high elaboration increased vividness and intentions to interact with outgroup members in the future. Furthermore, vividness predicted willingness to interact while controlling for changes in attitude and anxiety. The more vivid an imagined event was, the greater the willingness to interact.

Interestingly, the qualities of the event that were manipulated for the purposes of elaboration closely resemble defining features of episodic experiences: contextual and temporal specificity (Tulving, 2002). Based on studies of episodic memory and imagination that have found greater hippocampal activity when more vivid and detailed events are subjectively experienced (Addis et al., 2004; Addis and Schacter, 2008), an intriguing possibility is that the hippocampus may instantiate the effect of vividness on intergroup cognition.

In regards to memory and intergroup relations, Husnu and Crisp (2010) found that – irrespective of the elaboration condition – remembered past experiences positively predicted vividness of imagined events and willingness to interact with outgroup members in the future. This makes theoretical sense if, when constructing imagined events, bits and bobs of episodic details are gleaned across memories. Broadly consistent with the theories on imagination and memory (Ingvar, 1979; Tulving, 1985; Schacter and Addis, 2007), memory appears to support – or at least to enrich – imagination. And, it would seem, in the service of prosocial behavior. It is worth noting, however, that the imagined contact literature has relied heavily on self-reported measures of prosocial behavior, causing some to question the practical significance of these measures (Bigler and Hughes, 2010). This emerging literature awaits further experiments using objective behavioral measures. Yet, evidence from Turner and Crisp (2010) finding positive changes in implicit prejudice reduces some of these concerns (Crisp and Turner, 2010). Thus far, I have largely focused on psychological evidence that indicates a cognitive relationship between memory, imagination, and empathy. Further support comes from emerging evidence of a shared neural architecture.

## A Shared Neural Basis

The neuroscience on memory and imagination has developed largely independently from the neuroscience on social cognition (broadly) and empathy (specifically). Although initial findings from brain-damaged amnesic patients suggest that brain regions supporting memory and imagination may not be *necessary* to complete some mentalizing tasks (Rosenbaum et al., 2007), work from various clinical populations (e.g., Lombardo et al., 2007) and neuroimaging studies demonstrate that brain systems supporting memory and imagination may *shape* empathy. Demonstrating a link between memory for personal experiences and empathy, Lombardo et al. (2007) found that differences in self-referential memory between autistic patients and healthy controls disappeared when measures of empathy were included as a covariate (see also Corcoran and Frith, 2003; Lee et al., 2004 for co-morbid deficits in schizophrenia). Conversely, independence across these mental processes also exists. For example, older adults show diminished abilities to remember the past and imagine the future (Addis et al., 2008; Gaesser et al., 2011), yet they exhibit preserved levels of trait empathy across the lifespan (Gruhn et al., 2008). These behavioral findings underscore a complex relationship between processes, suggesting that memory and imagination may contribute to, but are distinct from, a capacity for empathy.

Here, I will briefly examine preliminary neuroimaging findings that are beginning to subvert theoretical autonomy across these processes, shedding light on a shared constellation of brain regions within the default network, recruited for remembering and imagining specific personal experiences as well as understanding and empathizing with others. To the extent that the brain is functionally localized, this anatomical overlap represents a cognitive interaction across faculties, signifying contributions of one faculty to another or component mental processes recruited by all faculties (Henson, 2005; Bressler and McIntosh, 2007; Hein and Knight, 2008). These findings raise more questions than they answer and pave the way for a promising new line of research.

While functional neuroimaging studies have provided initial evidence of the neural overlap between memory, imagination, and social cognition in the default network (Spreng et al., 2009; Spreng and Grady, 2010; Spreng and Mar, 2012), unexamined in these studies is the neural relationship of memory and imagination with empathic concern and action. However, recent neuroimaging studies are beginning to uncover commonalities.

Investigating the neural conditions that facilitate prosocial thoughts and behavior, Masten et al. (2011) found that regions of the default network were more strongly activated when subjects viewed social exclusion (i.e., a person in need) during a ball-tossing game compared to when they viewed social inclusion. Greater activity was observed for the mPFC and precuneus when subjects viewed social exclusion compared to inclusion. Further, after controlling for trait empathy levels in an exploratory mediation analysis, only activity in the mPFC positively predicted prosocial behavior (e.g., consoling the excluded player outside of the scanner; see also Rameson et al., 2012; Waytz et al., 2012).

Investigating the neural substrates of an enhanced empathy bias toward ingroup members relative to outgroup members, Mathur et al. (2010) showed that, across individuals, the difference in mPFC activity when observing someone in need (e.g., in a natural disaster) for ingroup versus outgroup members predicts state empathy and willingness to donate money and time to help ingroup members. Future work may want to investigate whether instructions similar to those used in the aforementioned imagined contact literature would boost mPFC activity and subsequent helping behavior.

One possible explanation for the involvement of the mPFC across these studies on empathy comes from its role in self-referential processing (Amodio and Frith, 2006). Psychological research has previously demonstrated that subjects who view a person in need as more similar to themselves show heightened empathetic concern for the similar-to-self person in need (Cialdini et al., 1997). Neuroimaging studies show the mPFC is preferentially activated when mentalizing about similar and psychologically close others (Krienen et al., 2010; Tamir and Mitchell, 2011; Denny et al., 2012). Therefore, mPFC activity may support empathy to the extent that it reflects a perceived self-other overlap, as greater mPFC activity may indicate an increase in perceived self-other similarity (Masten et al., 2011; Rabin and Rosenbaum, 2012).

The preceding studies typically interpret activity within the default network as a proxy of mentalizing processing. However, because similar regions are activated under conditions of episodic simulation (Schacter et al., 2008), it could be the case that this activity represents constructing vivid scenarios rather than simulating thoughts and feelings in some cases or perhaps shared component processes, such as self-referential processing. Further investigation is needed to adequately tease apart the entangled relationship across these cognitive processes in order to characterize the independent or interactive contributions of episodic simulation and mentalizing to prosocial cognition and behavior.

## Conclusion and Future Directions

Humans are a preeminent evolutionary success story because we work as a horde, a cognitively sophisticated and helpful horde. Multiple cognitive tools are thought to support our prosocial tendencies (Tomasello, 2000; de Waal, 2008). Prominent among these tools are the capacity to infer others’ mental states and an empathic emotional response to others’ plights (Batson, 1991). Here I have outlined the speculative possibility that episodic simulation of specific vivid and coherent scenarios may also foster helping behavior. Suggestive evidence from imagination inflation, imagined contact, and the neuroscience of memory, imagination, and empathy hints at a socially enhancing function for constructive memory and imagination.

To be clear, I am not proposing the adaptive function of promoting prosocial behavior to be mutually exclusive with other proposed functions of episodic simulation (e.g., “trying out” alternative scenarios in order to plan and predict the future). Nor am I advocating that episodic simulation will always be associated with prosocial behavior, but rather make the more modest claim that it may support prosocial behavior in healthy individuals and that promoting prosocial behavior may constitute the primary advantage (Nowak and Highfield, 2011). It is likely that imagination will only be beneficial to the extent that these simulations accurately reflect reality. And, while simulations of events can often be error prone (Dunning, 2007; Gilbert and Wilson, 2007), I am encouraged by a recent meta-analysis highlighting the relative accuracy of simulations (Mathieu and Gosling, 2012). There may also be particular circumstances in which episodic simulation could in fact reduce prosocial behavior. If the content of the simulation is focused on negative consequences associated with helping, such as damage to the self, then simulation may reduce the likelihood of intervening. Whether episodic simulation *can* be used to promote prosocial behavior, and whether episodic simulation *normally* promotes prosocial behavior are open questions of applied and theoretical relevance.

Future research is needed to establish the parameters under which episodic simulation may influence empathy and prosocial behavior. Of theoretical importance will be delineating the contributions of episodic simulation from known mental processes that influence prosocial behavior (e.g., mentalizing and affect-sharing). It may be the case that episodic simulation facilitates helping as a result of interacting with perspective taking or affect-sharing. For example, adopting a person in need’s perspective may very well guide imagining how to appropriately help someone, or perhaps the facilitating effect of episodic simulation will be partially attributed to making more cognitively available the perspective of another person’s thoughts and feelings. However, these interactions are unlikely to fully account for an increase in willingness to help to the extent that the effect of imagination inflation, which holds for non-social events, and the effect of increased temporal and contextual specificity of an event (both of which are definitional qualities of episodic simulation but not of mentalizing or affect-sharing) improving intentions to interact with outgroup members extend to helping behavior.

Identifying the precise degree of independence and interaction between the scenario construction and self-referential aspects of episodic simulation from traditional accounts of fostering empathy is paramount to determining the contribution of episodic simulation to prosocial behavior. An interesting question is whether episodic simulation could boost prosocial behavior in the absence of the capacity for perspective taking. One possibility is that semantic knowledge or episodic memories of past related helping experiences could be used to guide or inform episodic simulations of helping events without relying on perspective taking. For instances, perhaps the semantic knowledge that slow elderly people typically benefit from help crossing roads triggers an episodic simulation of how best to accompany a specific person upon observing an elderly person, cane in hand, waiting on a street corner, thereby promoting prosocial behavior.

Mechanistically, it remains unknown whether the effects of imagination will be primarily mediated by vividness and detail of the scenario (possibly substantiated in the hippocampus and precuneus), or by a self-other identity merging (possibly substantiated in the mPFC), or by some combination of the two. Specifying the effect of emotion is also of interest. Although mentalizing is well known to promote prosocial behavior by increasing emotional concern for others, there is currently no empirical evidence that the facilitating influence of imagining would also operate through emotional concern, but this does not dismiss a role for emotion in some capacity mediating the influence of simulation on prosocial behavior. Indeed, the increase in perceived likelihood that an imagined event will occur was restricted to emotional events in Szpunar and Schacter (2012) and imagining a positive social tone has been integral to eliciting imagined contact effects (Crisp and Turner, 2009).

At this point we are left to speculate and await empirical investigation to substantiate and critically evaluate the ideas outlined in this article. To the extent that a goal of society is to maximally foster these social abilities, identifying and examining all the mental processes that influence empathy and prosocial behavior is critical. Psychology and neuroscience have already illuminated several cognitive strategies for fostering empathy and prosocial behavior. But I wonder if perhaps humans are equipped with at least one more empathic tool: episodic simulation.

## Conflict of Interest Statement

The author declares that the research was conducted in the absence of any commercial or financial relationships that could be construed as a potential conflict of interest.
